# A missing color area extraction approach from high-resolution statue images for cultural heritage documentation

**DOI:** 10.1038/s41598-020-78254-w

**Published:** 2020-12-14

**Authors:** Adel Nasri, XianFeng Huang

**Affiliations:** State Key Laboratory of Information Engineering in Surveying, Mapping, and Remote Sensing, Wuhan University, Wuhan, 430079 China

**Keywords:** Engineering, Materials science

## Abstract

Ancient statues are usually fragile and have a tendency to deteriorate over time, developing cracks, corrosion, and losing color. Before any intervention on the object of art, a conservator must map degradation and take measurements. Deterioration mapping is an extremely long process, as the conservator or restorer must locate and digitize the damages manually and collect physical measurements from the artwork. Extracting and measuring the deterioration automatically from images is less expensive and aids the digital documentation process, thus reducing the time cost of manual deterioration mapping. In this paper, we propose an effective approach named Missing Color Area Extraction in order to extract and measure missing color areas from high-resolution imagery statues, using a thresholding technique. The conversion from RGB color space to HSV color space is applied, in addition to morphological operations to remove the dust and small objects.

## Introduction

Cultural Heritage is the most common mean of identity in any country. These heritage objects are often fragile, vulnerable, and sometimes even threatened to disappear for various reasons^1^. There is no doubt that the mission of transmitting cultural heritage to future generations is not limited to publish scientific studies data. If the heritage object as a material entity has value for the scientist in its historical significance, it remains the last trace and the last witness of our predecessor’s passage. Therefore, this legacy must be treated with respect for our human conscience, since non-renewable destruction irreversibly alienates our cultural heritage. We must make sure that they are well documented because the loss of heritage means the loss of a part of our identity.

According to^2^, cultural heritage conservation is considered a vital process in cultural heritage^3^. Additionally, the documentation process consists of three stages of understanding the importance, policy development, and management. In the fundamental needs of any conservation project and before planning any intervention in an asset of heritage interest, it would be better to have the most complete documentation possible and preferably in digital format to facilitate the management and sharing of the available information. Such documentation corresponds to the current state of the asset but should ideally continue in later phases to assist in monitoring and maintenance tasks. It is hard to obtain such documentation, but it is necessary to help preserve and disseminate tangible cultural heritage.

The pictures captured in many areas, such as earth science^4^, astronomy^5^, biology^6^, industry^7^, etc. helps to solve many of the issues that are hard to solve with traditional methods^8^. Images have also helped to develop important fields, such as heritage, which allows the assessment and measure of damaged areas without any physical contact^9^. Hence the rapid development of digital computing has led to a vast expansion of applications for computer vision systems^10–12^. Before, these processes need to be done manually. These processes are considered very tedious and costly such as feature identification, measurement, and cartographic elaboration. Nowadays, the automated process of feature extractions uses high-performance computers and cameras.

The relationship between cultural heritage and new technologies is very complicated^13^, and it requires a deep understanding and knowledge from multiple subjects. The use of image segmentation is demonstrating to be of prodigious help in the analysis and archival of heritage documentation^14^. There are many studies and published articles in recent years that propose algorithms to analyze painting images, where researchers provide some specific measurable appearances for cracks detection and removal in digital painting^15^, as well as for virtual restoration^16^.

Many ancient artworks, especially statues of Mogao Caves in Dunhuang temple, get damaged during the time and suffer from several degradations such as cracks, lacunas, and missing color. The missing Color area is the common damage that refers to the type of degradation and presents the pattern of original color changing that develops across ancient artwork. The missing color area phenomenon is due to the materials used for the artwork and the atmospheric variations that statues have been exposed to. In addition, other reasons such as physical tensions in the structure^17^, external mechanical factors^18^ such as human manipulation or storage conditions^19^.

The operation of degradation mapping, measuring, and monitoring cultural heritage is done manually in this field. Such a process is considered long and tedious due to cost and time. Therefore, the use of automatic methods that exploit high-resolution images represents a potentially interesting alternative.

Image Segmentation is a process comprising in separating an image into groups of items. The segmentation focuses on the creation of homogeneous colored regions characteristics^20^. These characteristics are generally defined in computer vision by discontinuities and similarities between intensity values, spectral radiation, or textural patterns within an image^21^. The result is a collection of pixel clusters where characteristics differ significantly from those of their neighbors in each cluster. The objective of segmentation is to add structure to the data, which allows a faster enabling and more accurate analysis. The application of image segmentation strategy in the heritage domain can be used in order to extract missing color regions^22^. The knowledge on the exact position or precise volume of missing color and risk areas is capital for conservators to elaborate the degradation mapping, initiate restoration operations by facilitating the delineation of regions of interest, visualizing, assessment, and measuring damaged areas.

In the field of heritage, most research studies focus on extracting, measuring, and removing of cracks from image paintings. However, in the literature, we found that the extraction and measurement of damaged areas in statues are not well explored. The images taken by cameras are often processed using different segmentation strategies^23^. A methodology measurement using a non-contact device (a CMOS digital) used by^23^. One creative program, for example, based on segmenting the decay zones from images of stone materials^24–26^. Also An integrated and automated segmentation approach to deteriorated regions recognition for cultural heritage artifacts^27,28^.

Overall, diverse approaches for image processing in the field of cultural heritage information extraction were proposed. The studied papers show that different techniques are used for many kinds of heritage applications^29^, and they gave different results. Therefore, the proposed methods are not efficient for all applications.

In this paper, we propose an approach of missing color area extraction measurement for ancient statues of Mogao Caves in Dunhuang, and we name Missing Color area extraction (for short, MCAE). In addition to a collection of different statue images downloaded from different sources. The main objective of this paper is to develop an overall digital image processing algorithm for automatic missing color area extraction and measurement from statues imagery, which indicates a damaged area on the statues, in addition, helping conservators and restorers to locate and determine precisely the measurement deterioration and its characteristics, as well as for the elaboration of degradation mapping. Moreover, this paper will take advantage not only as document archive and preservation purposes, but also for maintenance, rehabilitation, and restoration.

In this paper, we present two main contributions. This research is the first study that has been used for the extraction and measurement of damaged areas from high-resolution statue images for cultural heritage, where there is a lack of studies in this research area. Secondly, we propose MCAE, a simple and efficient methodology for the extraction and measurement of the missing color areas.

## Materials and methods

The ever -increasing value of images and computer vision techniques needs many demands for analysis, and automatic extracting and measuring of information using image segmentation to facilitate and improve the documentation, protection, and restoration process. This paper focuses on the use of images taken by digital cameras.

### Data

In this paper, the datasets provided by Daspatial company for academic research purposes were used in order to achieve our aim. There are two RGB images of statues of Mogao Caves in Dunhuang in this dataset. Mogao Caves is considered one of the most important World Cultural Heritage properties in China according to its archaeological data. The Mogao Caves are a unique site of outstanding value,and they represent an irreplaceable and irreproducible human resource. These caves are inscribed on the World Heritage list since 1987^30^, where we can observe several frescos, mural paintings, and precious statues dated from the fourth to the fifth century, which are located in 492 caves^31^. The Mogao Caves provides and documents precious and exceptional data for numerous fields such as history, art, and archaeology studies. In addition, the provided data helps to understand the cultural exchange and connection between West and South Asia during the antiquity. After the abandonment of the Silk Route in the thirteenth century, the caves were uninhabited, and the archeological data such as many statues, have deteriorated. The data provided by Daspatial company are two RGB images with a dimension of 5616 × 7344 taken by the Canon EOS-1Ds Mark III Camera.

Besides, various RGB statue images are downloaded from different websites. These images were captured with different cameras under different conditions, with different sizes. It is important to mention that the obtention of high-resolution statue images of damaged areas is not easy. As known, the accessibility to cultural heritage data is so limited due to the privacy and data protection issues.

#### Missing color area extraction

**Figure 1:**
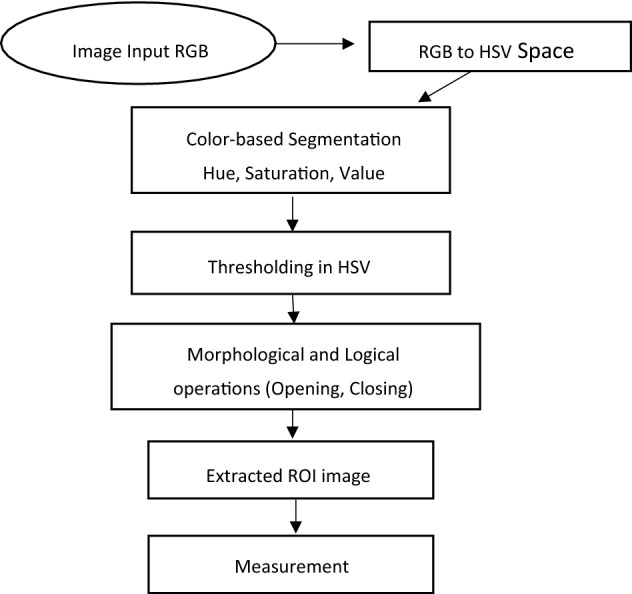
Illustrates the flowchart of the proposed approach for missing color area extraction.

Image processing methods for missing color area extraction for heritage protection is the term used in this paper. MCAE is defined as a procedure to find the similar region of interest in the pictures. MCAE is based on the similarities in their features, an 8-bit RGB image is the starting point, and the final result is supposed to be a binary image of missing color. In addition to different segmentation techniques, the extraction of missing color area involves the conversion between two model spaces, from RGB to HSV model space, Color-Based segmentation by using the thresholding technique of each component in HSV model space is investigated, in addition to logical and morphological operations. Figure 1 illustrates the main steps of MCAE approach.

We discuss the different steps used in the proposed approach MCAE in detail.

### RGB to HSV model space

The color range that a camera can see is described in a color space^32^. The purpose of defining a color model should be qualified and specified in some colors accepted normal manner. It is a collection of codes for every color. Each pixel in a picture has a color in the color space so that pixel labeling can use this color space. All colors can be defined in different ways, so there are diverse color spaces, as well. The size of a color space depends on the main color's number of tones^33^.

**Figure 2:**
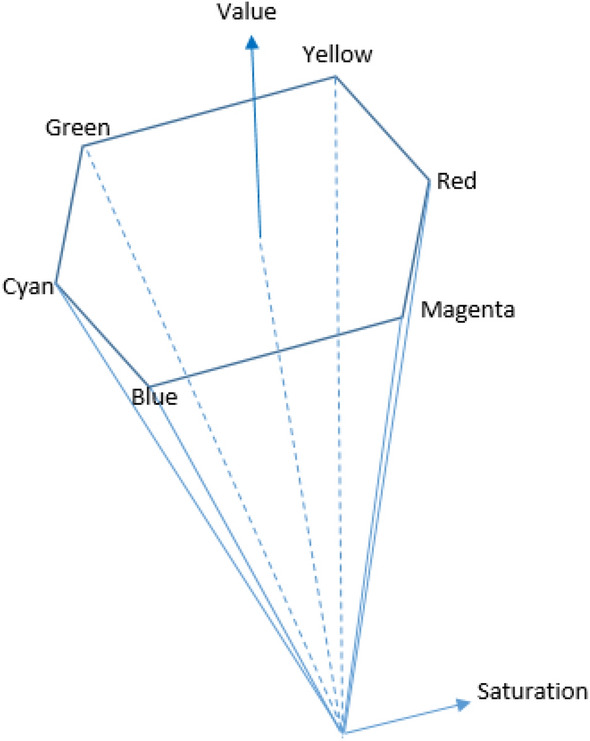
Illustration of the HSV color space.

The HSV coordinate system presented in^34^ is based on a hex-cone model, as it is shown in Fig. 2^33^. Hue, Saturation, and Value (HSV) model is widely used for developing image processing algorithms. The Hue is a color attribute that describes the pure color, Saturation is the measure of the degree to which the color described by Hue is diluted by white light, and value is the measure of intensity.

HSV is preferred over the red, green, and blue (RGB) for processing purposes because of its effectiveness in describing the color and for feature extraction, and illumination invariance separates the image intensity from the color information and reduces the effect of light changing.

There are three values of R, G, B. These values should be between 0 and 1. Each value range from 0 to 255, we divide each value first by 255.

The conversion from RGB model space to HSV model space is done by using the following set of equations:

The V band of each RGB pixel is given by the Eq. (1) used in^35^:

1 $$\mathrm{V}=\mathrm{max}\left\{\mathrm{R},\mathrm{G},\mathrm{B}\right\}$$

The S band is calculated by using the Eq. (2),

2 $$\mathrm{S }= \left\{ \frac{\mathrm{V}-\mathrm{min}\left(\mathrm{R},\mathrm{G},\mathrm{B}\right)}{\mathrm{V }-\mathrm{ max}(\mathrm{R},\mathrm{G},\mathrm{B}}\right\},\mathrm{ V}\ne 0$$

$$\mathrm{If\, S }= 0,\mathrm{ then\, H }= 0.$$

$$\mathrm{If\, R }=\mathrm{ V},$$

then, the H band is obtained by using the following equations:

3 $$H=\left\{\begin{array}{c}\frac{60\left(\mathrm{G }-\mathrm{ B}\right)}{\mathrm{V }-\mathrm{Min}\left(\mathrm{R},\mathrm{G},\mathrm{B}\right)}, G\ge B,\\ 360 +\frac{60(\mathrm{G }-\mathrm{ B})}{\mathrm{V }-\mathrm{ Min}(\mathrm{R},\mathrm{G},\mathrm{B})}, G< B,\end{array}\right.$$

If G = V, then,

4 $$H=120+\frac{60(\mathrm{B }-\mathrm{ R})}{\mathrm{V }-\mathrm{ Min}(\mathrm{R},\mathrm{G},\mathrm{B})} ,$$

If B = V, then,

5 $$H=240+\frac{60 * (\mathrm{R }-\mathrm{ B})}{\mathrm{V }-\mathrm{ Min}(\mathrm{R},\mathrm{G},\mathrm{B})},$$

For RGB image, it is necessary to have three separate elements of data for each pixel, a (true) color image of size $\mathrm{m}\times \mathrm{n}$ is represented by a matrix of extent $\mathrm{m}\times \mathrm{n}\times 3,$ which is considered a three-dimensional matrix. Considered such a matrix as a single unit containing three separate arrays which are aligned vertically. By using the colon operator, we can separate each color band. This step consists of extracting and computing each component individually.

### Color image segmentation

One of the techniques to do Image Segmentation is thresholding, which is to transform gray or RGB images into a binary image. The optimal measurement distance will be very important because consistency depends on the measurement of distances between colors. In this case, all methods found in the related works use the Euclidean distance to calculate the resemblance between two color pixels.

From the high-resolution image to do image-based color segmentation, region segmentation has a significant influence in order to extract the missing color area. Basically, color clustering is based on region. It colors the same area with tags to make the region of interest totally extracted and faster to use clustering to do segmentation work. It is also a kind of region-based image segmentation that can use the spatial characteristics to classify the pixel because it has a high sensitivity to color. According to the color distance, we cluster the similar colors. The distance is considered to the color close degree. As shown in Eq. (6):

6 $$\mathrm{F}=\sqrt{{{(\mathrm{H}}_{1}{-\mathrm{H}}_{2})}^{2}+{{(\mathrm{S}}_{1}{-\mathrm{S}}_{2})}^{2}+{{(\mathrm{V}}_{1}{-\mathrm{V}}_{2})}^{2}}$$

where H, S, V are image color components, respectively.

After clustering the similar colors, an operation of selecting typical samples of area patterns and get color distance about samples and area on the image. Indeed, the dust is making things a bit harder because the color of region of interest (ROI) and the dust on the statue have very close pixel values. Based on the typical color of an element as the cluster center, if the distance is less than the set threshold $Th$, it estimates the pixel approximately as region pixel of the image, then it compares the results of distance to the thresholds until the major elements of the image are all carrying out color clustering.

Based on the color distance formula for color clustering, the threshold has a great effect on the results of clustering. Threshold formula is in Eq. (7):

7 $$\sqrt{({\mathrm{D}1)}^{2}+{(\mathrm{D}2)}^{2}+{(\mathrm{D}3)}^{2}}<\mathrm{Th}$$

where D1, D2, D3 are the values after averaging between the given color patch and image elements. After color clustering, the missing color area is extracted.

### Mathematical morphology

After the thresholding step, mask those thresholding values of each color content for each color in the image by using logical operators. Then we combine the masks of three bands to find where all three bands are "true.", then we will have the mask only the region of interest (ROI).

The logical operators are changed according to the color of the missing area. Mathematical morphology and filtrate were first introduced by^36^ and^37^ for geometric analysis. The image’s morphology operation^38^ is one of the most significant tools used in digital image processing used by researchers in various scientific fields. Based on a formal framework of mathematics, mathematical morphology delivers a method to the processing of digital images that are based on geometrical shape. It uses operations such as dilation, erosion, openings, closings. The dilation of set A by B is defined in Eq. (8):

8 $$A\oplus B= \left\{z\left|{\widehat{\left(B\right)}}_{z }\cap A \subseteq A\right.\right\}.$$

where z is all the displacements of the origin of B over A, where at least one element in B ^ extends beyond the elements in A. Erosion of set A by B is defined in Eq. (9):

9 $$A\ominus B= \left\{z\left|{(B)}_{z }\subseteq A \right.\right\}.$$

The erosion of A and B process is similar to dilation, except that the boundaries of A are reduced instead of enlarged. The process performs a relative complement operation on A by B, for all z.

There are many types of morphological closing operation based on shapes, each pixel in the image is adjusted based on the value of other pixels in its neighborhood. By choosing the size and shape of the neighborhood.

As the dust has shown as a missing color area, the opening operation can successfully clean and remove high-intensity forms that are smaller in width and/or length than the structuring element (SE)^39^. As well as, the closing operation, which is the combination between dilation and erosion, is efficient in smoothing contour portions, eliminate low-intensity forms that are smaller in width and/or length than the structuring element and fill gaps in the contour. As it is mentioned above, there is a must to measure the extracted region due to this reason. The gaps in all these sets must be filled so that the measures obtained are reliable.

A logical AND operation is achieved after masking the color content between those masked color contents to extract the region of interest of our images. The output of this step has resulted in an image with the representation out of the area of interest. Before the final step, the resulted image of logical AND operated in the previous step, we multiply operation with the original RGB image.

Since the RGB-space is a standard color space, an imported image has its RGB-values automatically. Each pixel in the image has its own value of red, green, and blue, and these values define a color matrix coordinate. At this coordinate, the pixel will receive the color matrix value. We have labeled each blob extracted in order to get the measurement of the mean HSV and the extracted area of our region of interest (ROI).

## Results and discussion

We describe all processing tasks with the output results in this section. The proposed method is tested on two data sets: the first dataset is two RGB images 1 and 2 of statues of Mogao Caves in Dunhuang.

In the second dataset, we use our proposed approach on 18 RGB statue images from 3 to 20 of heritage statues downloaded from different sources. The size of selected images differs from an image to another, and the image varies in brightness, background, size of the damaged area, and characteristics.

The implementation and processing were performed in MATLAB 2015b. All experiments were performed on a personal computer with an Intel Xeon CPU E3-1240 v5 processor with 3.50 GHz and 16 GB RAM.

**Figure 3:**
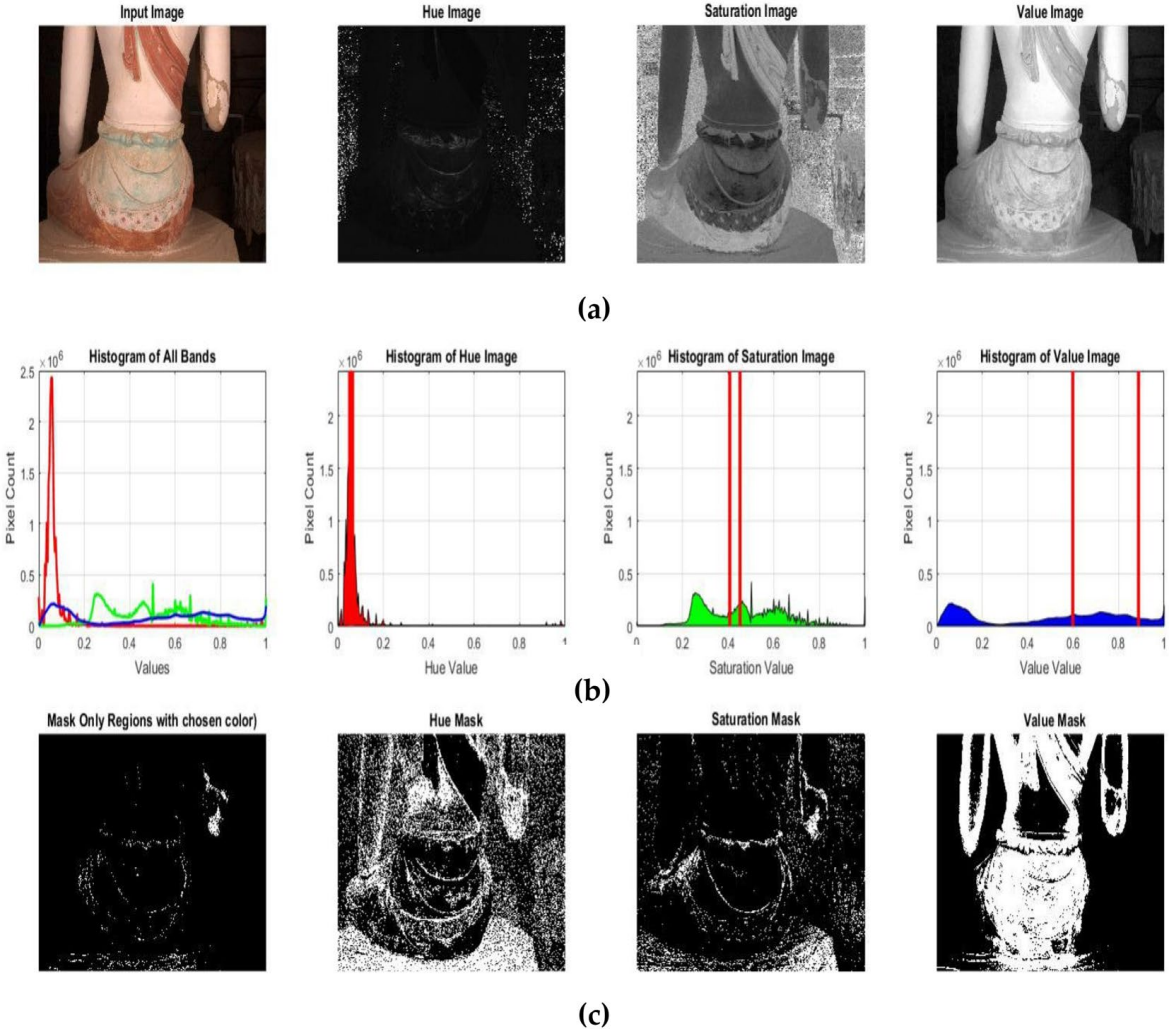
(**a**) Image input; histograms of hue, saturation, and value, (**b**) histogram of all bands and histogram of hue, saturation and value, each histogram shows the perfect thresholding ranges found based on MCAE and distribution of colors in the image. (**c**) Mask only regions with the chosen color, mask of hue, saturation and value.

By using our proposed approach color-based segmentation MCAE, the case missing color area was separated from the low saturation of the other regions. To carry out this segmentation, several images are generated during the analysis of image 1 in Fig. 3.

The ranges of threshold ${Th}_{H}$ for a missing color area pixel of the three-color bands in HSV color space used by our algorithm are: 0.052 ≤  ${Th}_{H}$ ≤ 0.106 for Hue band, 0.407 ≤  ${Th}_{S}$≤ 0.452 for Saturation band and 0.598 ≤  ${Th}_{V}$≤ 0.887 for Value band.

**Figure 4:**
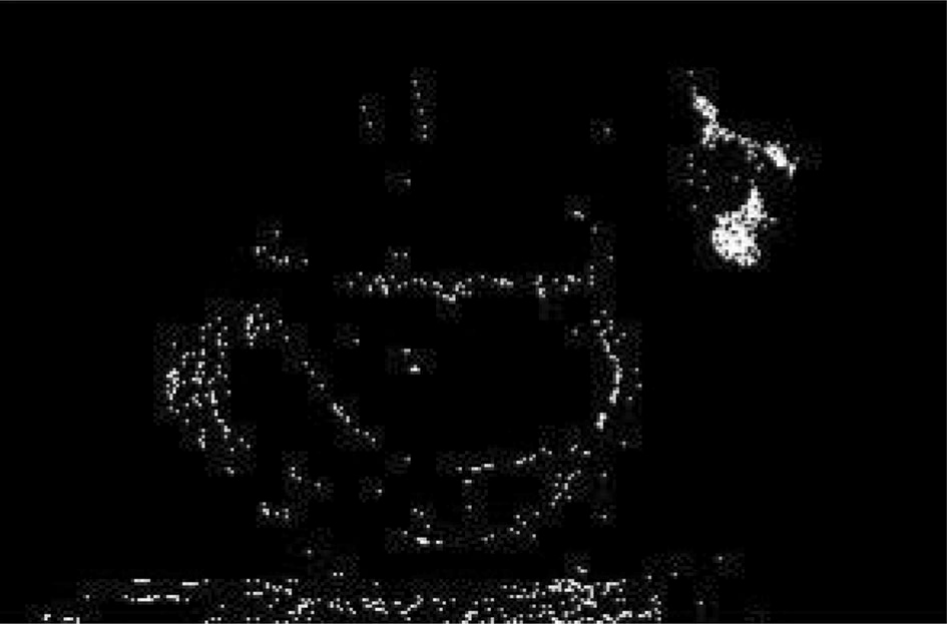
Result after segmentation shows the dust extracted as missing color area.

As it is shown in Fig. 4, we have obtained a fairly good image segmentation because dust is covered different parts of the statue, which is pretty close to the targeted region of interest. The pixel values of the dust and missing color area of the statue are very close to each other in the original image, which makes it difficult to extract only the missing color region of the statue. Those pixels are associated with the wrong region of interest (ROI). We have cleaned the dust and other small objects (< 5000 pixels) by opening operation and by chooing a disk structuring element with a diameter of 4.

Note that the variations of those values result in the different selected images give different results. For each image, the size of missing color area, dust, the level of details, and brightness of each image.

Furthermore, closing operation used for smoothing the border completes all the lost gapes in the statue as they are most likely also extracted.

**Figure 5:**
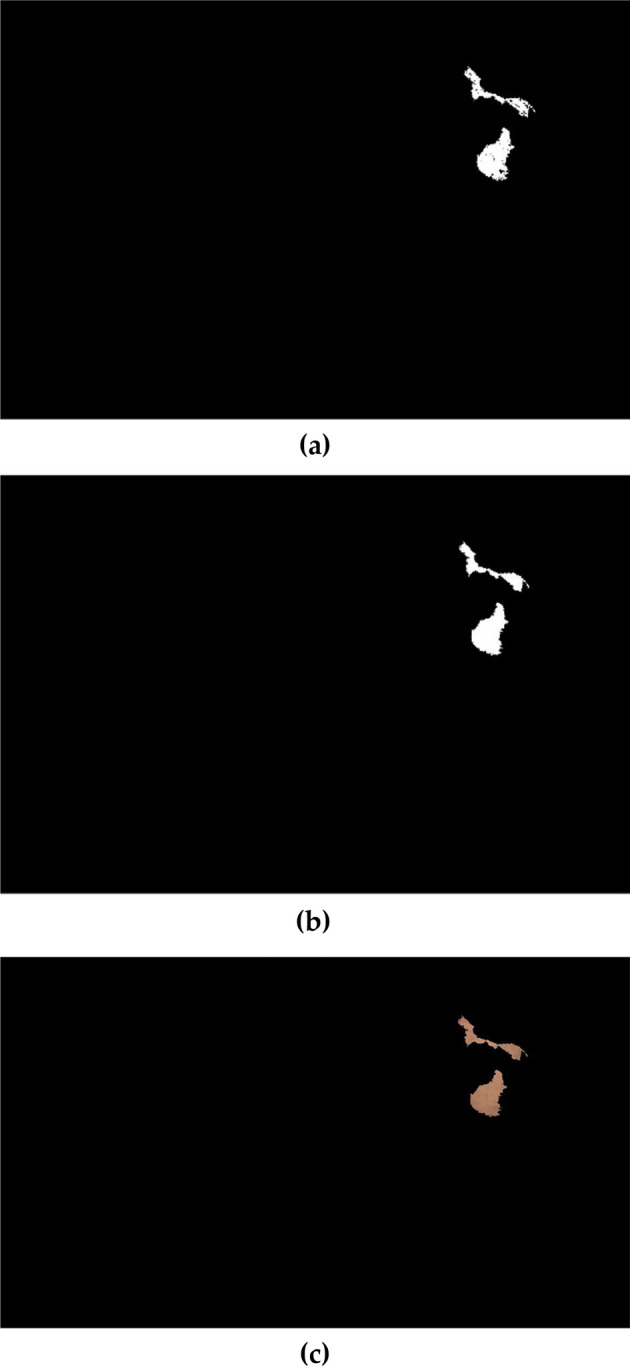
The obtained results of image 1. (**a**) Border smoothed, (**b**) region filled, (**c**) The big particle region of missing color area extracted.

**Figure 6:**
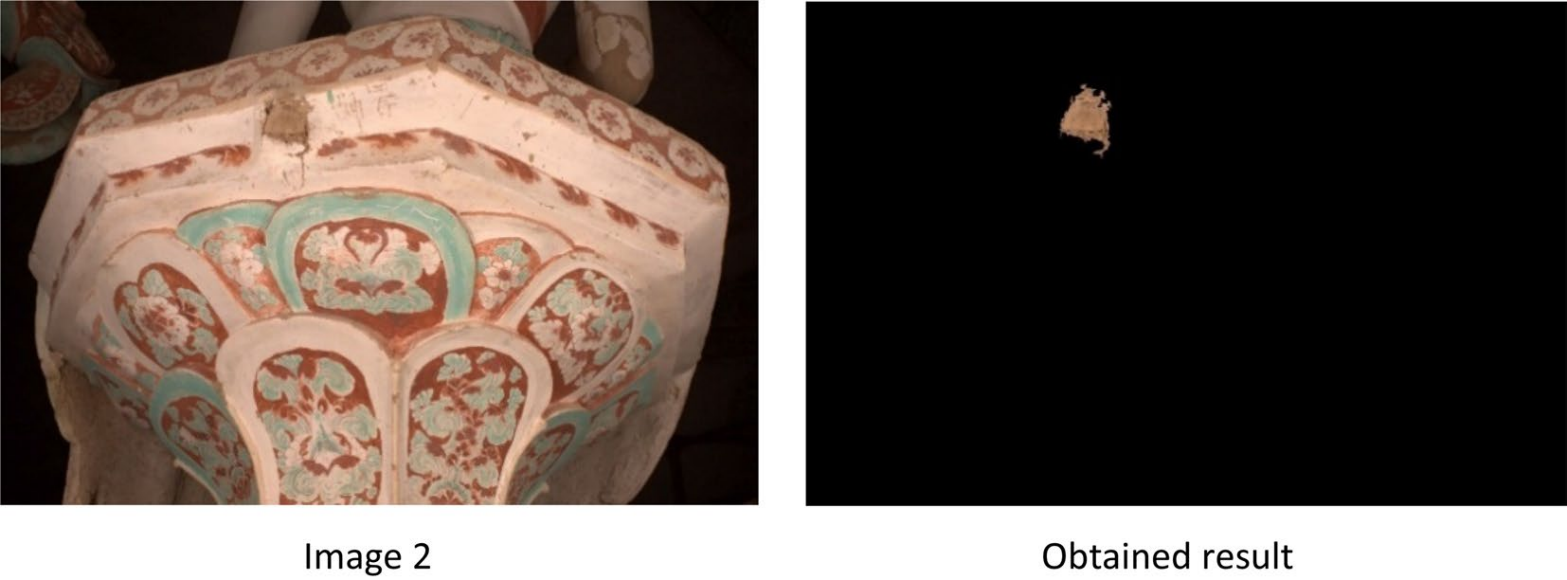
Shows the experimental results on the statue base of Mogao Caves. To extract the ROI, we set the parameters as follows: 0.058 ≤  ${Th}_{H}$ ≤ 0.106 for hue band, 0.416 ≤  ${Th}_{S}$≤ 0.662 for saturation band and 0.539 ≤  ${Th}_{V}$≤ 0.734 for the value band. Opening operation (< 2000 pixels) and by choosing a disk structuring element with a diameter of 4.

**Figure 7:**
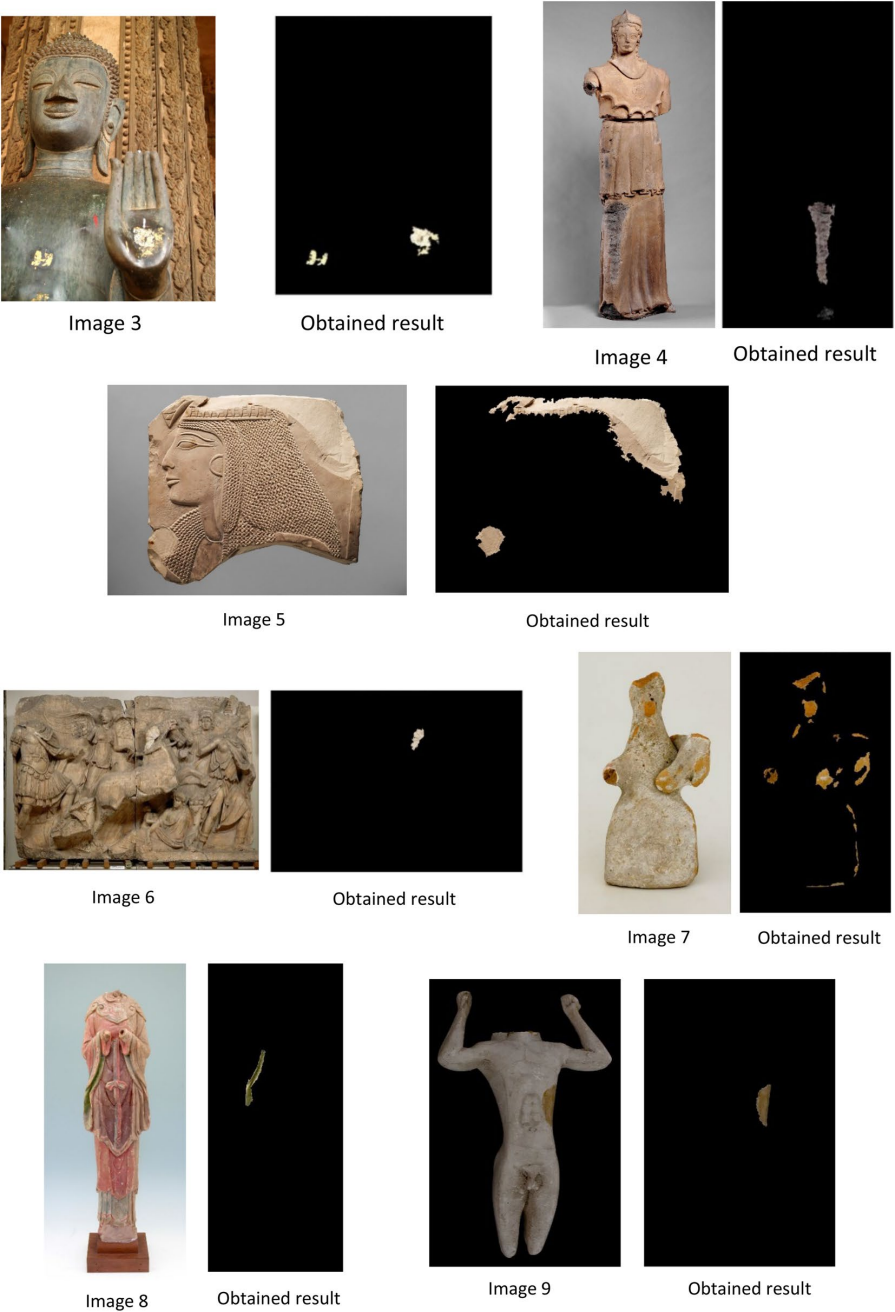

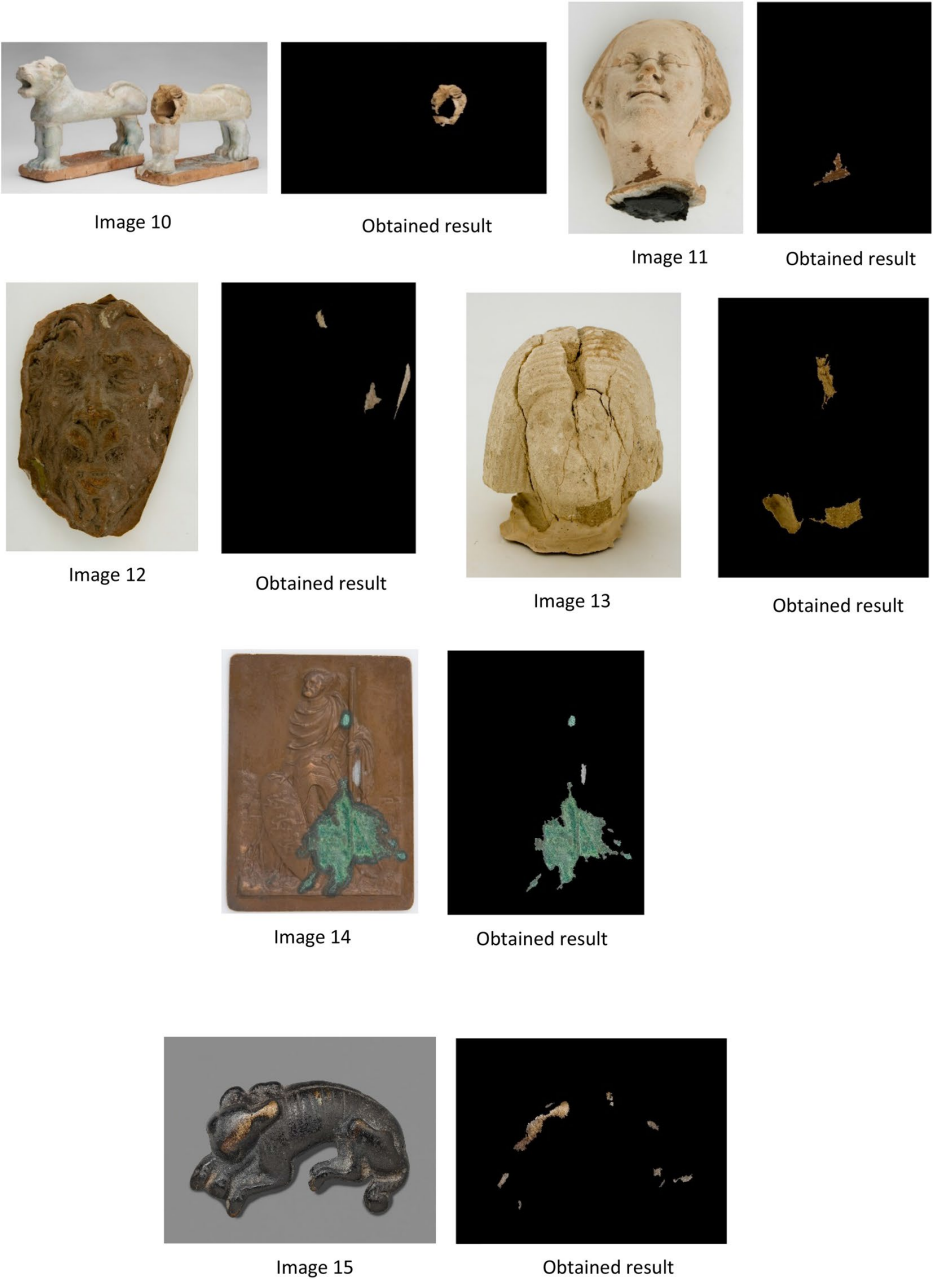

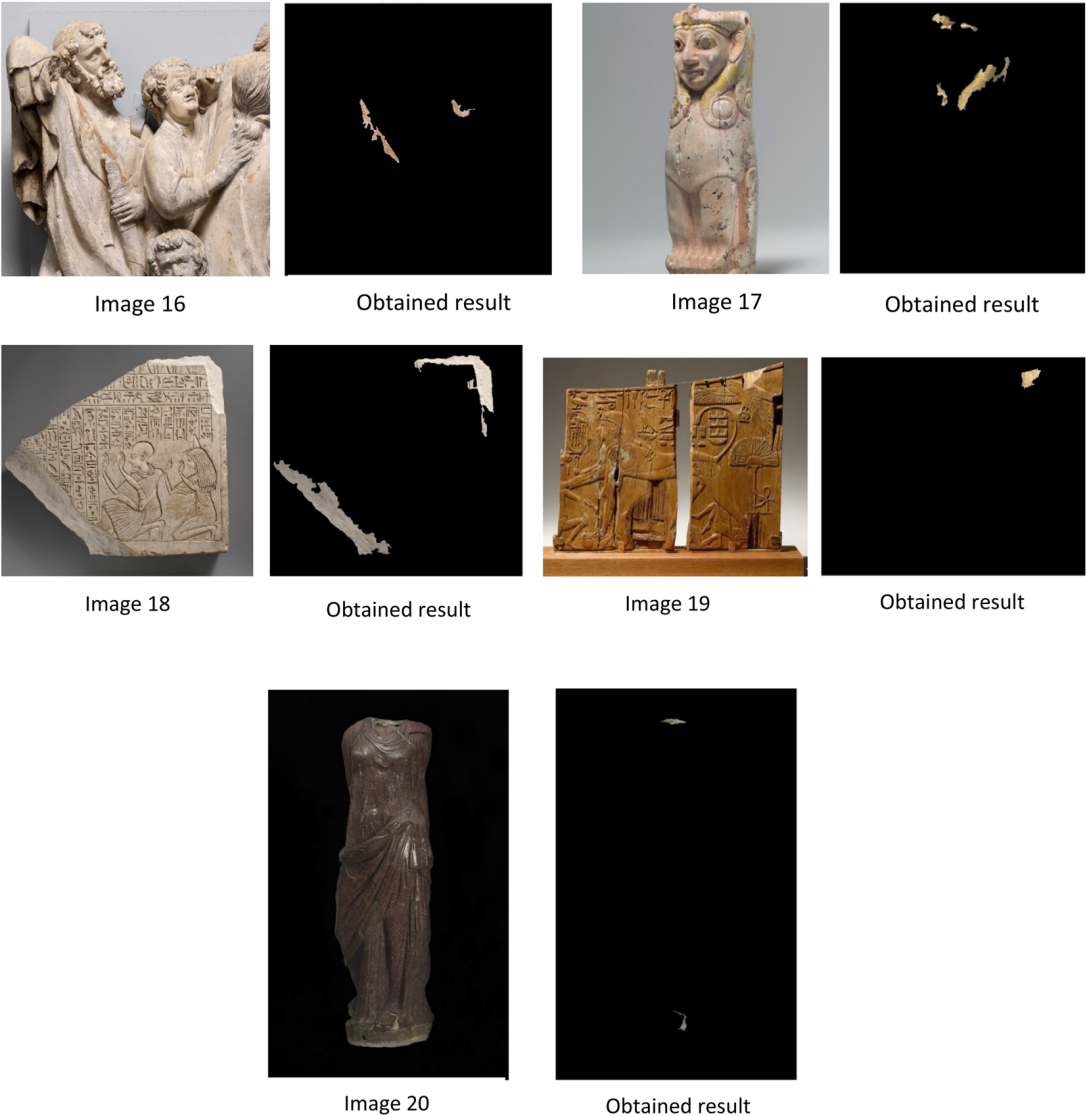
Shows the images used and the output of applying MCAE approach.

The last results obtained of image 1 and 2 are shown in Figs. 5 and 6 respectively:

According to the results shown in Fig. 7, missing color regions are extracted successfully by using our proposed approach MCAE. The measurement of the extracted areas to get a number of pixels extracted shown in Table 1.

**Table 1:** Measurement of the extracted regions by using our proposed MCAE approach.

Image	Number of pixels measured
1	135,146
2	104,901
3	45,597
4	114,275
5	81,189
6	21,004
7	19,688
8	3343
9	5001
10	13,696
11	6251
12	8479
13	28,198
14	58,527
15	65,954
16	5328
17	9513
18	41,787
19	3102
20	1365

After applying the proposed approach MCAE on the dataset of twenty statue images of missing color areas, the results obtained were as follows: The use of the MCAE approach gives an outstanding output that measured missing color areas. Also, a visual check and examination by eye demonstrate that the proposed automatic extraction method based-color successes to extract and measure the desired missing color areas. Nevertheless, the accuracy assessment of this result must be completed in order to assess the efficiency of the proposed method.

The quantitative evaluation of the experimental results is achieved by comparing the results of MCAE against a manually digitized using ImageJ, which is a cross-platform, open-source image processing and analysis software used in all scientific fields^40^. However, imageJ particularly focuses on biomedical applications. In our case, ImageJ is used to get the total number of pixels, this tool is considered a high-quality reference model to perform the accuracy as summarized in Table 2, for the quantitative evaluation of the obtained results.

**Table 2:** Summary of accuracy measurement between our proposed approach MCAE and IMAJ J.

Image	Number of pixels measured Using MCAE	Number of pixels measured reference model IMAJ J
1	135,146	168,655
2	104,901	106,171
3	45,597	46,816
4	114,275	122,483
5	81,189	87,177
6	21,004	23,333
7	19,688	21,127
8	3343	3520
9	5001	5061
10	13,696	14,863
11	6251	7146
12	8479	9315
13	28,198	30,220
14	58,527	59,640
15	65,954	70,527
16	5328	6013
17	9513	10,720
18	41,787	55,916
19	3102	3755
20	1365	2080

From Table 2, the results of missing color area extraction MCAE are quite satisfied with an average rate of 90.38%, except image No 01 has a low measurement compared to the rest of the images. These results are due to the presence of the dust. The dust, the environmental lighting, and surface conditions of the statues have a significant influence on unmeasured missing color areas.

## Conclusions

This study mainly presents a method for automatic extraction and measurement of missing color area MCAE from statue images of cultural heritage. Firstly, conversion from RGB model space to HSV model space is needed. Then Color-Based segmentation is initiated based on the thresholding technique, which is followed by masking thresholding values. Moreover, we performed morphological opening and closing operations to remove dust and small objects from images and some other logical operations.

Based on the comparison of the extraction results with the digitization of missing color areas, the results of the proposed approach MCAE determine about 90% of the missing color areas are extracted, while about 10% of region of interest is unextracted due to the dust, variations of the lighting, surface conditions, and environment.

The important advantage of the proposed approach MCAE is considered in its simplicity and robustness that can be extended for the extraction and measurement of other types of pathologies in the heritage domain. This study shows the usefulness of an automated system as a low-cost alternative for manual degradation mapping and measuring its extracted regions.

However, as we mentioned above, many factors have a significant impact on measuring and extracting missing color areas, such as dust, environmental lighting, and surface conditions of the statues. Therefore the proposed method does not reach its best performance on some regions, in which one or more influenced factor is present. As future work, we plan to improve this work in several directions. First, we will deal with various factors and adapt the proposed method to different extraction targets by applying deep learning methods for identifying regions covered by dust. In addition, we plan to develop a method that enables a virtual restoration of missing color areas. Finally, we intend to extend our system for mural painting application.

## Data Availability

The datasets generated during and/or analyzed during the current study are available from the corresponding author on reasonable request.
